# Motor cortex modulation and reward in children with attention-deficit/hyperactivity disorder

**DOI:** 10.1093/braincomms/fcab093

**Published:** 2021-05-04

**Authors:** Jordan A Detrick, Caroline Zink, Keri Shiels Rosch, Paul S Horn, David A Huddleston, Deana Crocetti, Steve W Wu, Ernest V Pedapati, Eric M Wassermann, Stewart H Mostofsky, Donald L Gilbert

**Affiliations:** 1 University of Cincinnati College of Medicine, Cincinnati, OH, USA; 2 Division of Neurology, Department of Pediatrics, Cincinnati Children’s Hospital Medical Center, University of Cincinnati College of Medicine, Cincinnati, OH, USA; 3 Baltimore Research and Education Foundation, Baltimore, MD, USA; 4 Department of Psychiatry and Behavioral Sciences, Johns Hopkins University School of Medicine, Baltimore, MD, USA; 5 Lieber Institute for Brain Development, Baltimore, MD, USA; 6 Department of Neuropsychology, Center for Neurodevelopmental and Imaging Research, Kennedy Krieger Institute, Baltimore, MD, USA; 7 Department of Psychiatry, Cincinnati Children’s Hospital Medical Center, Cincinnati, OH, USA; 8 Behavioral Neurology Unit, National Institute of Neurological Disorders and Stroke, NIH, Bethesda, MD, USA; 9 Department of Neurology, Johns Hopkins University School of Medicine, Baltimore, MD, USA

**Keywords:** ADHD, transcranial magnetic stimulation, reward, motor cortex, children

## Abstract

Attention-deficit/hyperactivity disorder, the most prevalent developmental disorder in childhood, is a biologically heterogenous condition characterized by impaired attention and impulse control as well as motoric hyperactivity and anomalous motor skill development. Neuropsychological testing often demonstrates impairments in motivation and reward-related decision making in attention-deficit/hyperactivity disorder, believed to indicate dysfunction of the dopamine reward pathway. Development of reliable, non-invasive, easily obtained and quantitative biomarkers correlating with the presence and severity of clinical symptoms and impaired domains of function could aid in identifying meaningful attention-deficit/hyperactivity disorder subgroups and targeting appropriate treatments. To this end, 55 (37 male) 8–12-year-old children with attention-deficit/hyperactivity disorder and 50 (32 male) age-matched, typically-developing controls were enrolled in a transcranial magnetic stimulation protocol—used previously to quantify cortical disinhibition in both attention-deficit/hyperactivity disorder and Parkinson’s Disease—with a child-friendly reward motivation task. The primary outcomes were reward task-induced changes in short interval cortical inhibition and up-modulation of motor evoked potential amplitudes, evaluated using mixed model, repeated measure regression. Our results show that both reward cues and reward receipt reduce short-interval cortical inhibition, and that baseline differences by diagnosis (less inhibition in attention-deficit/hyperactivity disorder) were no longer present when reward was cued or received. Similarly, both reward cues and reward receipt up-modulated motor evoked potential amplitudes, but, differentiating the two groups, this Task-Related-Up-Modulation was decreased in children with attention-deficit/hyperactivity disorder. Furthermore, more severe hyperactive/impulsive symptoms correlated significantly with less up-modulation with success in obtaining reward. These results suggest that in children with attention-deficit/hyperactivity disorder, short interval cortical inhibition may reflect baseline deficiencies as well as processes that normalize performance under rewarded conditions. Task-Related-Up-Modulation may reflect general hypo-responsiveness in attention-deficit/hyperactivity disorder to both reward cue and, especially in more hyperactive/impulsive children, to successful reward receipt. These findings support transcranial magnetic stimulation evoked cortical inhibition and task-induced excitability as biomarkers of clinically relevant domains of dysfunction in childhood attention-deficit/hyperactivity disorder.

## Teaser

In this study of reward effects in motor cortex in children with attention-deficit/hyperactivity disorder (ADHD) compared to their typically developing peers, Detrick et al. report that transcranial magnetic stimulation-evoked short-interval cortical inhibition and task-related up-modulation significantly differed between groups and may therefore represent measurable biomarkers in ADHD.

## Introduction

Children diagnosed with Attention-Deficit Hyperactivity Disorder (ADHD) experience significant academic and social impairments and incur high medical costs.^1^^,^^2^ These outcomes may result in part from inefficient response inhibition and atypical response to reward.^3^^,^^4^ Improving childhood function may require moving past current practices, in which heterogeneous Diagnostic and Statistical Manual of Mental Disorders, 5th Edition diagnoses are based on subjective ratings, to development of quantitative, brain-based measures^5^ validated by determining their relationships to symptom severity, domains of dysfunction, and utility in targeting treatments. One appealing candidate for the development of brain-based measures is the motor system, based on well-characterized atypical features of motor development in ADHD.^6^

To investigate the motor system as a potential ADHD biomarker and quantify its engagement in relevant functional domains, we and others have employed Transcranial Magnetic Stimulation (TMS) to non-invasively evaluate motor cortex (M1) in children with ADHD and typically-developing controls.^7^ TMS generates electromagnetic fields that induce electrical currents in underlying neural tissue and, when administered over M1, can evoke a ‘motor evoked potential’ (MEP) in target muscle.^8^ The MEP amplitude reflects the TMS pulse intensity and the instantaneous excitability of M1. In *paired-pulse-TMS*, administering a conditioning TMS pulse activates inhibitory interneurons, reducing M1 excitability and thereby MEP amplitudes,^9^ as in paired-pulse TMS short interval cortical inhibition (SICI).^10^ Prior paired-pulse-TMS studies have shown that ADHD children have reduced SICI,^11^^,^^12^ that greater ADHD severity correlates with more reduced SICI,^12^^,^^13^ and that ADHD medications affect SICI.^14^^,^^15^ In *functional-TMS*, administering TMS during performance of a functional circuit that includes M1 may up-modulate MEP amplitudes,^16^^,^^17^ which we term ‘Task-Related Up-Modulation’ (TRUM). Prior functional-TMS studies have shown that ADHD children have reduced TRUM during a response inhibition task.^18^

The present investigation was designed to evaluate SICI and TRUM as they relate to the domain of reward valence in children with ADHD. Altered responses to reward cues and outcomes may contribute to long-term risk-taking behaviour and addiction in ADHD.^19^^,^^20^ The rationale for SICI as a putative reward-biomarker is that anomalous responses to reward in ADHD^21–23^ are believed to reflect abnormalities in the dopamine-reward pathway,^24^ and both SICI and dopamine are deficient in Parkinson’s Disease.^25^ Furthermore, experimental studies manipulating reward contingencies have shown that performance-based rewards improve performance in children with ADHD,^23^^,^^26^ and behavioural and pharmacological treatments use reward-based contingencies^27^ and increase dopamine availability.^28^

The overlapping and integrated neural circuitry governing inhibitory and reward processes^29^ suggest that, similar to response inhibition findings,^18^ reward may also differentially modulate SICI and TRUM in children with ADHD. Additional rationales for evaluating M1 during reward tasks include: M1 neurons have dopamine receptors,^30^ dopaminergic drugs modify excitability of M1 as assessed by TMS,^31^ and dopaminergic projections to M1 mediate motor skill learning.^32^ Furthermore, reward expectation modulates neural spiking and local field potentials in M1,^33^ and inhibiting M1 impairs learning under rewarded conditions.^34^

Finally, the present paediatric ADHD study builds on studies in adults using TMS during reward paradigms.^35–37^ Of particular interest, a study of SICI in a reward probability paradigm showed modulation of SICI occurred in healthy adults but did not occur in adults with mild Parkinson Disease in the unmedicated (low dopamine) state.^37^ Healthy adults also show up-modulation in M1 excitability in anticipation of desired rewards.^38^ We anticipated, based on these studies, that reward paradigms in healthy children might also induce changes in M1 SICI and TRUM, but that, similar to adults with Parkinson Disease, in unmedicated children with ADHD, such reward-induced changes might be diminished. Therefore, we undertook this study to examine whether reward-predicting cues and reward-receipt modulate SICI and induce TRUM in children, whether reward-induced changes in SICI or TRUM are diminished in ADHD (categorical diagnosis), and whether such changes correlate with the ADHD severity. Although these studies using TMS, as well as the present study, do not directly measure dopamine, we speculate that by combining TMS with a reward task we may gain insights to processes believed to be dopamine related.

## Materials and methods

### Subjects

This study compares M1 physiology and reaction times (RTs) during a reward task in 105 children, ages 8–12 years: 55 ADHD; 50 Typically Developing (TD) controls. Analyses were performed both with ADHD as a categorical diagnosis and with parent-rated ADHD symptoms as a dimension of behaviour.

### Standard protocol approvals, registrations and patient consents

All participants were recruited concurrently from two urban medical centers via advertisements, from 2011 to 2017. Written informed consent was obtained from the legal guardians of study participants. The study was approved by the Johns Hopkins Medicine and Cincinnati Children’s Hospital Medical Center Institutional Review Boards.

### Clinical assessments of cases and controls

For ADHD evaluation, the ADHD Rating Scale IV (ADHD-RS-IV)^39^ and the Conners Parent Rating Scale (CPRS)-Revised^40^ or third edition^41^ were used. Children with ADHD were included based on diagnostic thresholds on both scales, confirmed using structured diagnostic interviews^42^^,^^43^ by paediatric neurologists (DLG, SHM). TD Children did not meet diagnostic criteria for any psychiatric diagnosis. Additional exclusion criteria for both groups included: neurological illness/injury, seizures, left-handedness as assessed by the Edinburgh Handedness Inventory,^44^ full scale intelligence quotient < 80^45^^,^^46^ and dyslexia. Home environment was evaluated with the Hollingshead Parent History Questionnaire.^47^ Psychostimulants, but no other medications, were allowed, but were temporarily discontinued the day prior to testing.

### Transcranial magnetic stimulation: measure of resting (baseline) motor cortex physiology

Study personnel trained together for consistency. Both sites used Magstim 200^®^ transcranial magnetic stimulators (TMS) (Magstim Co., New York, NY, USA) connected through a Bistim^®^ module to a round 90 mm coil, as well as identical amplifiers, filter settings, disposable surface electrodes, and Cambridge Electronic Design 1401 and Signal^®^ 6 software for monitoring responses in real time and automated off-line extraction of peak-to-peak MEP amplitudes for analysis. We recorded EMG (electromyelography) from the relaxed first dorsal interosseous. All EMG tracings were observed in signal in real time and participants were reminded to maintain a fully relaxed hand. Trials with visible artefact were tagged and removed offline. The TMS coil was placed flat near the vertex, with coil-current direction counterclockwise to optimally evoke MEPs. All protocols implemented by our laboratories in hyperkinetic children have been previously described,^12^ for motor thresholds [active motor threshold (AMT), resting motor threshold (RMT)],^48^ cortical silent periods (CSP),^49^ SICI and intracortical facilitation (ICF).^10^ In brief, thresholds—minimum %MSO required to evoke an MEP peak-to-peak amplitude of ≥0.05 mV in 3 of 6 trials at rest (RMT) and above background activity during tonic muscle contraction (AMT); CSP—rectified average duration of EMG suppression during 5 trials, TMS pulses 1.5*AMT, during sustained tonic muscle contraction; paired TMS—conditioning pulses at 0.6*RMT, test pulses at 1.2*RMT; SICI—3 ms inter-stimulus intervals; ICF—10 ms inter-stimulus intervals, 10 trials each randomized with 10 single pulse, with an intertrial interval of 6 s ± 10%.

### ‘Moneybags Game’ reward paradigm

**Figure 1:**
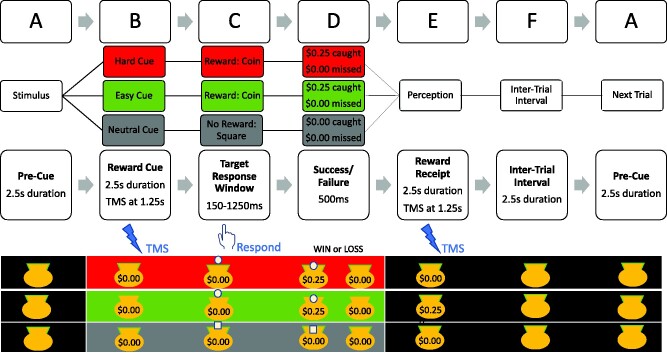
**Schema of ‘Moneybags’ Reward Game.** The $0.25 coin or the non-reward square chip appears at the onset of segment C. TMS probes occur at midpoints for segments B (cue) and E (reward receipt). The participant action in response to the coin or chip occurs in segment D. Red Cues indicate probability of success is 1 in 3, Green Cues indicate probability is 2 in 3. See Materials and Methods.

TMS was administered during the Money Bags Task (see Fig. 1) in children with ADHD and TD controls to evaluate whether this reward task differentially (i) up-modulates motor cortex excitability (Task-Related-Up-Modulation, or TRUM) and/or (ii) alters SICI. This task^50^ is based on the widely used monetary incentive delay paradigm^51^ and incorporates the Expectancy Theory of Motivation.^52^ Participants faced a computer monitor running the task in Presentation^®^ (v. 10.0, Neurobehavioral Systems, Albany, CA) while comfortably seated with ulnar aspects of both upper limbs on a body-surrounding pillow. The dominant right hand index finger operated a computer mouse. The program presented a ‘money bag’ with a background colour that cues the child about whether a reward may be achievable (25-cent coin; red or green background) or not (non-coin square-chip; grey background). Participants were instructed through a standardized script that their job is to ‘catch’ the coin or chip before it disappeared into the bag to earn it at the end of the game. To do this, they must respond to the coin/chip *as quickly as possible* by pressing the mouse button. Participants were also informed that it is more difficult to ‘win’ the 25-cent coin in red-background trials because the coin disappears more quickly than in green trials. Trials were administered in pseudo-randomized order. Successes were tracked with cumulative earnings displayed on the game screen. No money was added or subtracted for a failure-to-catch or any grey-background trials.

In two-thirds of red-cue trials and one-third of green-cue trials, the coin disappeared in 150 ms, ensuring failure but giving participants the impression that they were not quite fast enough to succeed. Conversely, in two-thirds of green-cue and one-third of red-cue trials, the coin remained on the screen until they push the button, for up to 1250 ms, ensuring success while giving the perception the reward was due to their effort/fast response time. Grey-cues were evenly divided between fast and slow disappearance. The red-cue thus should have elicited highest motivation. Reaction results supporting the theory of this paradigm^50^ would be Red (fastest) < Green < Grey (slowest). A similar ordering of cue_SICI or cue_TRUM would support that motivation is reflected in these biomarkers. The action/TMS-pulse interval of >200 ms was designed to avoid EMG/MEP contamination by movement preparation.

Red, Green and Grey-Cue Trials occurred at a 1:1:1 ratio. For the first 35 participants, the game included only TMS during the Cue-phase: 96 trials (32 of each cue type). As children tolerated this procedure without requiring breaks, for the remaining 70 participants the number of trials was increased from 96 to 186 and pulse timing was randomized to occur during the cue or receipt phases (see Fig. 1). Finally, for the 70 participants, after a short break, to explore whether reward-related physiological changes might depend on movement, a 32-trial version was administered in which participants experienced the same cues, rewards, and TMS pulses but were instructed not to move/respond when the coin or chip appeared, unless the cue was framed in yellow (2 trials), the purpose of which was to verify ongoing attention only.

### Statistical analysis

#### Univariate analyses of behaviour, motor function, motor physiology and clinical/demographic variables

Motor, physiological, behavioural and demographic data were compared across diagnostic groups using *t*-tests and Chi Square as appropriate. For ‘method of means’ analyses, SICI and ICF are expressed as ratios of average paired to average single pulse-evoked, MEP amplitudes.

#### Repeated measures analyses of MEP data

All models were analysed using SAS^®^ version 9.4 (SAS Institute Inc., Cary, NC), using repeated measures mixed models with subject as a random effect. For SICI and TRUM (M1 physiology), MEP amplitude is the dependent variable; for behaviour, RT is the dependent variable. ADHD diagnosis was evaluated primarily as a categorical variable and secondarily as a dimensional variable based on Conners-scale parent-ratings *T*-scores. Age, Sex and Site were included in all models. A *P* value less than 0.05 was considered significant.

Increased MEP amplitudes may result from increased excitatory, reduced inhibitory (disinhibition), or some combination of inputs into M1. Faster RTs may result from greater motivation for a reward, related to perceived difficulty. We aimed to determine whether SICI, TRUM and RTs were associated with diagnosis, symptom severity, cue type (red, green, grey), or reward receipt (success, failure), or their interactions.

#### Reaction time

RT is the ‘behavioural output’ of this task.^52^ Mean RTs and variability were compared between groups. In order to improve characteristics of linear modelling, the dependent variable RTs were transformed to their natural logarithm. To evaluate the relationships of RT to the Reward Task and diagnosis, the models evaluated these hypotheses: (i) RT differences for cue—Red, Green, Grey; (ii) RT differences across diagnostic groups—ADHD, TD; (iii) RT relationship to ADHD severity—worse ADHD symptoms, slower RTs; and (iv) diagnosis and RT differences across cues—cue*diagnosis interaction. To explore the relationship between RT and M1 physiology, models were also created to assess: (i) associations of RT and the amplitude of the MEP during the trial and (ii) association of RTs with baseline M1 physiological variables (RMT, AMT, CSP, SICI, ICF).

#### Short interval cortical inhibition

After evaluation of the standard model residuals showed heteroscedasticity, MEP amplitudes were log transformed. The dependent variable was ln(MEP amplitude). In these models, SICI was estimated from the ratio of the Least Squares Means estimates for PulseType (3 ms paired pulse numerator; single pulse denominator). The models evaluated: (i) the relationship between SICI and Cue and Receipt—hypotheses: both higher reward probability (green cue) and reward receipt (success), would be associated with increased SICI; (ii) SICI differences across diagnostic groups—hypotheses: the effects of Cue and Receipt on SICI would be diminished in ADHD; and (iii) the relationship between SICI and ADHD symptom severity—hypotheses: the effects of Cue and Receipt on SICI would be more diminished with more severe ADHD. These hypotheses were evaluated using all trials, both reward task and rest, then *post hoc* these hypotheses were evaluated within the full task and within the sections of the task.

#### Task-related up-modulation

TRUM was estimated based on ratios of the Least Square Means estimates for Task [task MEP amplitude numerator; baseline (rest) MEP amplitude denominator]. A higher ratio above 1.0 indicates more TRUM. The models evaluated: (i) the relationship between TRUM and Cue and Receipt—hypotheses: higher reward probability (green cue), and reward receipt (success), would be associated with increased TRUM; (ii) TRUM differences across diagnostic groups—hypotheses: the effects of Cue and Receipt on TRUM would be diminished in ADHD; and (iii) the relationship between TRUM and ADHD symptom severity—hypotheses: the effects of Cue and Receipt on TRUM would be more diminished with more severe ADHD.

Additional exploratory analyses were performed by including in the models baseline physiological measures (RMT, AMT, CSP, ICF, MEP amplitudes).

### Data availability

The data supporting the findings of this study are available, anonymized for any reasonable request.

## Results

### Subject demographics

Demographic, clinical and physiological variables were compared by group (see Table 1). The recruited sample included 105 right-handed children (55 ADHD, 50 TD controls; 36 Cincinnati; 69 Baltimore). As expected, ratings for ADHD symptoms were significantly greater in ADHD for all scales.

**Table 1:** Demographics, clinical ratings, and physiology: Group comparisons

	ADHD			Typically developing			*P*-value
	*n*	%		*n*	%		
**Demographics**							
Site							0.005
CCHMC	12	21.8		24	48.0		
KKI	43	78.2		26	52.0		
Sex							0.72
Female	18	32.7		18	36.0		
Male	37	67.3		32	64.0		
Race							0.0003
African American	14	25.5		4	8.0		
Asian	1	1.8		5	10.0		
Biracial	8	14.5		6	12.0		
Caucasian	32	58.2		35	70.0		

	***n***	**Mean**	**SD**	***n***	**Mean**	**SD**	***P*-value**

Age	55	10.6	1.3	50	10.4	1.3	0.49
IQ Testing							
GAI	48	105.3	12.5	46	117.2	17.5	0.0003
ADHD severity scales—Conners							
Inattentive T score	52	75.6	10.7	48	46.9	8.2	<0.0001
Hyperactive/impulsive T Score	52	74.3	15.0	48	47.9	8.1	<0.0001
Resting physiology—TMS							
3 ms pair MEP mean (mV)	49	0.37	0.23	48	0.29	0.27	0.14
Single pulse MEP mean (mV)	49	0.66	0.31	48	0.60	0.34	0.38
SICI ratio^a^	49	0.61	0.39	48	0.47	0.25	0.05
ICF ratio	47	1.17	0.63	44	1.04	0.39	0.23
Resting motor threshold	54	60.4	9.4	50	64.5	10.4	0.04
Active motor threshold	52	44.0	8.1	49	44.8	7.7	0.61
Cortical silent period (ms)	42	58.9	41.8	44	71.0	46.3	0.21

Demographic characteristics, clinical ratings and resting M1 physiological measures in children with ADHD versus typically-developing controls. Categorical variables compared with Chi Square and continuous variables with *t*-tests. CCHMC, Cincinnati Children’s Hospital Medical Center; KKI, Kennedy Krieger Institute; GAI, General Ability Index; TMS, transcranial magnetic stimulation; MEP, motor evoked potential; mV, millivolt; SD, standard deviation; SICI, short interval (3 ms paired pulse) cortical inhibition (larger ratios indicate less inhibition); ICF, intracortical facilitation; motor thresholds (resting, active) as percentage of maximum stimulator output; ms millisecond.

a Estimate of SICI here is from the ‘method of means’ and differs from estimate derived from mixed model including all trials.

### Reaction time significantly slower in ADHD

Across cue types, RTs averaged 32–34 ms slower in ADHD versus TD children [*F*(1,133.4) = 10.0, *P *=* *0.02] (see Table 2). RTs were also slower in children with worse ADHD symptoms [Conners Inattentive *F*(1,125) = 7.97, *P *=* *0.0055; Conners Hyperactive/Impulsive *F*(1,125.7) = 15.3, *P *=* *0.0001]. RT variability was not significantly different between groups (*P *=* *0.68; Satterthwaite method).

**Table 2:** Reaction times by cue type and diagnosis

Trial type	Red	Green	Neutral
All			
ms	293	314	347
Ln	5.68	5.75	5.85
SE	0.02	0.02	0.02

Trial type*diagnosis			

ADHD			
ms	309	332	365
Ln	5.73	5.81	5.90
SE	0.03	0.03	0.03
Typically-developing			
ms	276	298	332
Ln	5.62	5.70	5.80
SE	0.03	0.03	0.03
*P*-value for diagnosis, within trial type	0.0048	0.0063	0.017

**Overall trial success**	**ADHD**	**TD**	***P*-value**

Logit (LSM)	0.439	0.403	
SE	0.03	0.03	
Odds	1.551	1.496	
Trial success %	60.8	59.9	0.41

Reaction times in Moneybag task. Red cues had lower success probability than green cues (see Fig. 1, Materials and Methods). Neutral cues had no reward. ADHD, children with attention-deficit/hyperactivity disorder; Ln, Natural log; LSM, least square means; Ms, milliseconds; SE, standard error; TD, typically-developing children. Mean reaction time estimates generated through exponentiation of regression estimates.

As shown previously in healthy adults performing this task,^50^ response times were fastest for Red/Coin and slowest for Grey/Chip across all participants [Cue Type *F*(2,18030) = 269.3, *P *<* *0.0001] (see Table 2). The Cue effect did not differ by diagnosis [Diagnosis*Cue interaction term *F*(2,18028) = 0.15, *P *=* *0.86]. As expected, older children had faster responses [*F*(1,133) = 39.85, *P *<* *0.0001], approximately 3% faster (slope estimate for log of RT = −0.03, SE 0.004) per each one year older.

**Figure 2:**
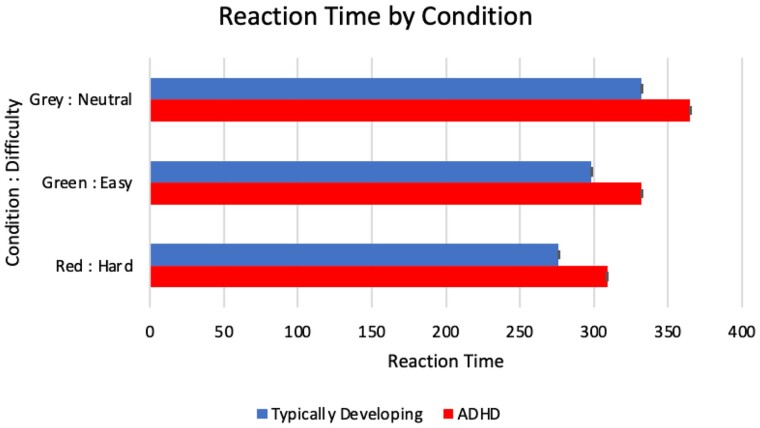
**Reaction times by cue type in ADHD versus TD children.** Reaction times in ms. Cue types were R, red (har difficulty, higher motivation, lower probability to catch coin); G, Green (easy difficulty; lower motivation, higher probability to catch coin); N, neutral grey (no reward possible, instructed to move as quickly as possible to catch the non-coin chip); ADHD, attention-deficit/hyperactivity disorder; TD, typically-developing control. Error bars are standard errors.

Larger MEP amplitudes were associated with faster RTs [*F*(1,13455) = 23.51, *P *<* *0.0001]. Baseline (rest) physiological measures were not associated with RTs (not shown). See Fig. 2.

### SICI is less in ADHD at rest

M1 physiology at rest is shown in Table 1. Calculated using the method of means (ratio of the average paired-to-single-pulse MEP amplitudes), the mean SICI ratio in ADHD was 0.61 (39% inhibition) versus 0.47 (53% inhibition) in TD children (*t*_95_ = 2.1, *P* = 0.05). RMT was higher in TD children (*t*_102_ = 2.1, *P* = 0.04). There were no other between-group physiological differences.

### Reward task reduces SICI in ADHD and TD

The All-Participants, initial model (Table 3, top) used MEP amplitude as the dependent variable and evaluated effects of task (rest versus all reward trials) and PulseType (for SICI). SICI ratios are calculated from LSMeans ratio estimates: 3 ms paired divided by single pulse MEP amplitudes. Compared to rest, task participation significantly increased MEP amplitudes [Task *F*(14,821) = 1409.68, *P *<* *0.0001]. SICI was significantly reduced (higher ratio) during the reward task compared to rest [PulseType*Task interaction *F*(1,14729) = 67.79, *P *<* *0.0001]. Stratifying by phases of the task, SICI reduction compared to rest was significant for both reward cue [PulseType*Task interaction *F*(1,9708) = 86.52, *P *<* *0.0001] and reward receipt [PulseType*Task interaction *F*(1,7041) = 28.15, *P *<* *0.0001].

**Table 3:** Motor cortex short interval cortical inhibition (SICI) in ADHD and TD children during rest, reward cue and reward receipt segments of the Moneybags task

		All participants					
		SICI ratio	SE		DF	*F*-value	*P*-value
Task*PulseType							
All Reward Task vs. Rest		0.67	0.05		14729	67.79	<0.0001
Cue vs. Rest		0.72	0.05		9708	86.52	<0.0001
Receipt vs. Rest		0.62	0.04		7041	28.15	<0.0001
Rest		0.55	0.05				*

	**ADHD**		**TD**				
	**SICI ratio**	**SE**	**SICI ratio**	**SE**	**DF**	***F*-value**	***P*-value**

PulseType*Dx							
Rest only	0.62	0.07	0.48	0.07	2022	19.20	<0.0001
Task*PulseType*Dx							
All Reward vs. Rest	0.71	0.07	0.67	0.06	14739	15.41	<0.0001
Post hoc, within all reward							
Cue phase only	0.74	0.08	0.71	0.08	8234	2.13	0.14
Receipt phase only	0.70	0.09	0.59	0.09	5614	1.80	0.18
Post hoc, within cue type							
Red	0.74	0.08	0.70	0.08	2596	0.00	0.95
Green	0.76	0.09	0.71	0.08	2895	0.98	0.32
Neutral	0.70	0.08	0.72	0.08	2544	3.95	0.05
Post hoc, within receipt outcome							
Failure	0.70	0.10	0.57	0.10	2243	0.62	0.43
Success	0.70	0.09	0.62	0.09	3301	1.72	0.19

**Figure 3:**
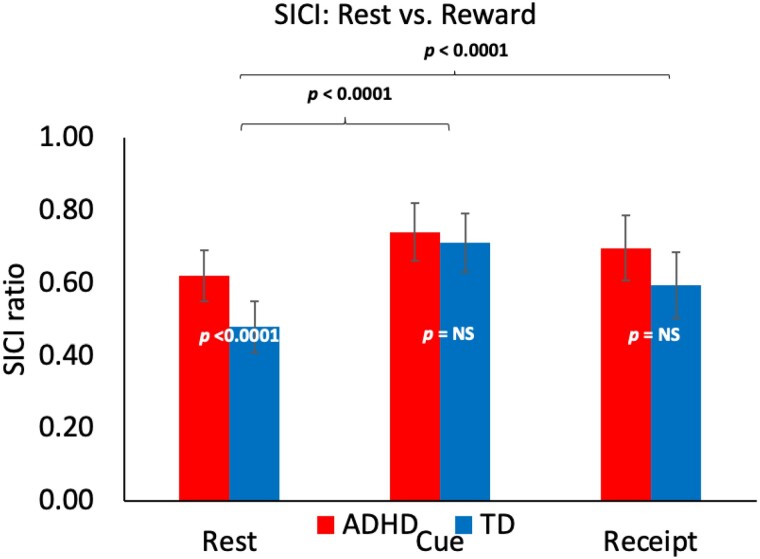
**Short interval cortical inhibition in ADHD versus TD children.** Figure shows post-hoc analyses stratified by task. See text, Table 3 for full regression results. Motor cortex (M1), TMS-evoked short interval cortical inhibition (SICI) ratios: conditioned (3 ms paired pulse) to test (single) pulse. A ratio of 1.0 indicates no inhibition; higher ratios therefore indicate less inhibition. At rest, children with ADHD have less SICI (higher ratios). During both Cue and Receipt, reward task participation significantly reduces SICI (ratios approach 1.0). Within the reward task segments of cue and receipt, SICI ratios for ADHD and TD do not differ (*P* = NS). From mixed models, LSMeans estimates: *P* values are from diagnosis (ADHD vs. TD) by PulseType (paired vs. single) interaction terms; error bars are standard errors of the mean (SEM).

Transcranial Magnetic Stimulation (TMS) motor evoked potential (MEP), short interval cortical inhibition (SICI) ratios (see Materials and Methods). Ratios closer to 1.0 indicate less inhibition. ADHD, attention-deficit/hyperactivity disorder; SE, standard error; TD, typically-developing children. SICI ratios are from Least Square Means from regression models (see text). Children with ADHD have less SICI at rest. Task is comprised of Cue and Reward Receipt segments. Cues are Red (low probability), Green (high probability), and Neutral (no probability); Rewards can be failures or successes. The all-participant model shows SICI reduction (compared to rest) during the reward task. The effect of the reward task on SICI is significantly greater in TD children, who start with more baseline SICI (see Fig. 3). Within the cue and receipt phases of the reward task (without considering rest), SICI does not differ between ADHD and TD children. Within the cue types, there is no difference by diagnosis when reward is possible, and a marginally significant difference by diagnosis in the neutral cue trials.

### SICI in ADHD subjects converged with that of TD subjects during the reward task

The categorical diagnosis models (Table 3, middle/bottom) evaluated interactions of diagnosis with SICI and reward task to determine if the Task/SICI findings differed for children with ADHD. Across all rest and reward trials, SICI was reduced in ADHD [PulseType*Dx interaction *F*(1,14739) = 9.16, *P *=* *0.0025] and during the reward task compared to rest [PulseType*Task interaction *F*(1,14739) = 67.81, *P *<* *0.0001]. This reward-task-change in SICI was significantly less in ADHD [PulseType*Dx*Task Interaction *F*(1,14739) = 15.41, *P *<* *0.0001]. However, the smaller rest→ task difference occurs because the SICI ratios differed at baseline (*P* < 0.0001) but converged during reward (See Fig. 3).

### SICI during reward task not robustly correlated with symptom severity

#### Across diagnoses

**Figure 4:**
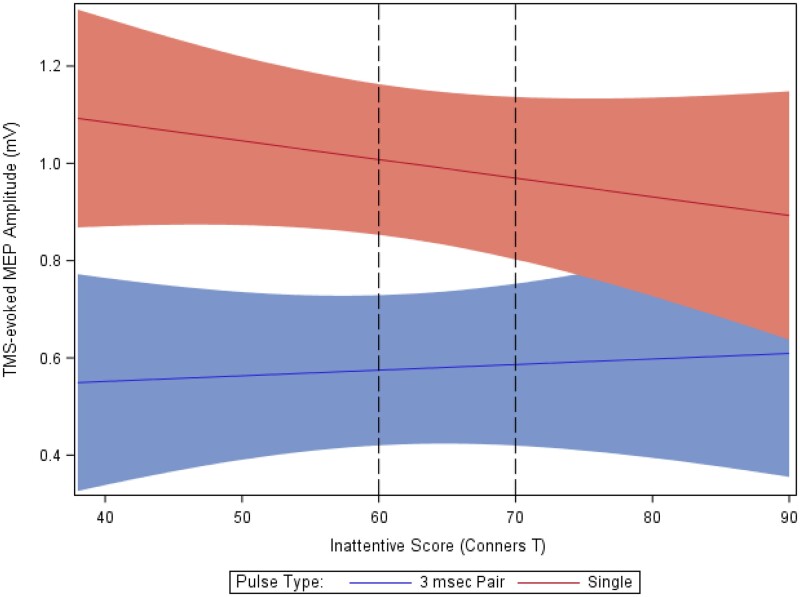
**Motor cortex inhibition during reward receipt phase, successful trials.** Motor cortex inhibition as a function of attention-deficit/hyperactivity disorder (ADHD) score severity based on Connors *T* scores for inattention. Association in success trials (reward-receipt) of less SICI with greater Inattentive Symptoms (*T* scores). T scores greater than 70 indicate high associations with ADHD, and below 60 with unaffected status. A score between 60 and 70 is intermediate. For the TMS MEPs, the upper, solid red line indicates the single-pulse (sp) transcranial magnetic stimulation (TMS)–evoked motor evoked potential (MEP) amplitudes during the receipt phase of the reward task, with the curves modelling the standard error. There were approximately 27 single pulse (upper, red) and 27 paired pulse trials (lower, blue) per participant. Less SICI is represented by less vertical ‘distance’ from the 3 ms to the single pulse. Worse ADHD severity is marginally associated with less SICI (*P *=* *0.05).

To determine whether there were greater reductions in SICI among children with more severe symptoms, the regression models for reward-cue and reward-receipt trials were re-run, replacing the categorical diagnoses with the ADHD Conners Scores. Analysing trials with TMS pulses during reward-receipt, SICI marginally achieved significance among children with higher inattentive scores after successful reward-receipt outcomes [*F*(1,3117) = 3.77, *P *=* *0.05] (greater inattention scores, less SICI, see Fig. 4) but not failure outcomes [*F*(1,2116) = 0.17, *P *=* *0.68]. Findings for children with higher hyperactive–impulsive scores were similar for both success [*F*(1,3116) = 0.47, *P *=* *0.49] and failure [*F*(1,2117) = 0.14, *P *=* *0.70]. Analysing trials with TMS pulses during reward-cue phase, we found no evidence of relationships between SICI and ADHD severity ratings.

#### Within diagnoses

Regression models were re-run separately with only TD and with only ADHD participants. During the task Cue phase, within the TD cohort, inattentive scores showed varying degrees of association with SICI. Higher inattentiveness was associated with less SICI during Green [*F*(1,1349) = 4.09; *P *=* *0.04] and Grey/Neutral [*F*(1,1196) = 9.15; *P *=* *0.003) trials, but not during red trials [*F*(1,1238) = 2.86; *P *=* *0.09). This association of less SICI with higher inattentive scores was found within ADHD children only during Green trials [*F*(1,1406) = 4.23; *P *=* *0.04]. Within Cue phase, higher hyperactive–impulsive scores were significantly correlated with less SICI in TD children during only Red trials (*F *=* *4.51; *P *=* *0.03), but were not associated with SICI in ADHD children in any cue. Within the Receipt phase, no associations were significant for SICI and inattentive nor hyperactive–impulsive scores.

### Reward task robustly increases motor cortex excitability (TRUM)

The All-Participants, initial model used MEP amplitude as the dependent variable and evaluated effects of task participation on TRUM. MEP amplitudes were significantly up-modulated during the task [Task effect *F*(1,14822) = 1400.01; *P *<* *0.0001]. Stratifying by phases of the task, TRUM was significantly increased from rest in both receipt phase [*F*(1,6999) = 971.78; *P *<* *0.0001) and cue phase [*F*(1,9811) = 1077.81; *P *<* *0.0001] (see Table 4).

**Table 4:** Motor cortex task-related up-modulation (TRUM) in ADHD and TD children during cue and receipt segments of reward task

		All participants					
		TRUM ratio	SE		DF	*F*-value	*P*-value
Task							
All Reward		2.06	0.05		14822	1400.01	<0.0001
Cue phase only		2.08	0.05		9811	1234.64	<0.0001
Receipt phase only		2.07	0.04		6999	971.78	<0.0001

	**ADHD**		**TD**				
	**TRUM ratio**	**SE**	**TRUM ratio**	**SE**	**DF**	***F*-value**	***P*-value**

Task*Dx							
All Reward	1.82	0.07	2.32	0.06	14819	75.82	<0.0001
Cue phase only	1.84	0.07	2.35	0.06	9822	70.2	<0.0001
Receipt phase only	1.80	0.06	2.36	0.05	5528	107.61	<0.0001
Post hoc, within cue type							
Red	1.89	0.05	2.36	0.05	4571	39.96	<0.0001
Green	1.94	0.06	2.41	0.05	4850	52.89	<0.0001
Neutral	1.77	0.05	2.34	0.05	4522	62.23	<0.0001
Post hoc, within receipt outcome							
Failure	1.90	0.05	2.49	0.05	4146	83.68	<0.0001
Success	1.75	0.05	2.29	0.05	5112	100.69	<0.0001

Task-related up-modulation of transcranial magnetic stimulation-evoked motor evoked potential amplitudes (see Materials and Methods). Ratios greater than 1.0 indicate greater up-modulation. ADHD, attention-deficit/hyperactivity disorder; SE, standard error; TD, typically-developing children. TRUM ratios are from Least Square Means from regression models (see text). Children with ADHD have less TRUM during all task segments. Task is comprised of Cue and Reward Receipt segments. Cues are Red (low probability), Green (high probability), and Neutral (no probability); Receipt can be failures or successes.

### TRUM significantly less in ADHD during all task phases

**Figure 5:**
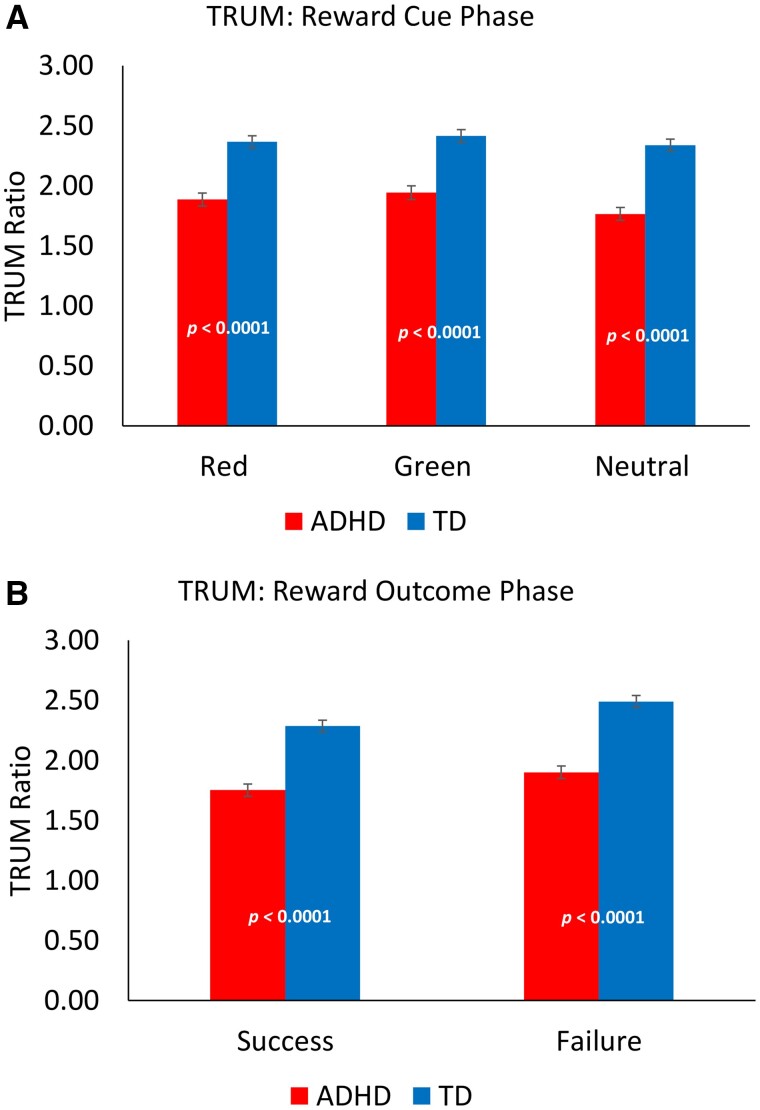
**Task-related up-modulation (TRUM) in ADHD and TD children.** Motor cortex (M1), TMS-evoked TRUM is a ratio of mean MEP amplitudes during task (or segment of task) compared to rest. A ratio of 1.0 indicates no up-modulation; higher ratios therefore indicate more TRUM. Across all cue types (**A**) and reward receipt outcomes (**B**), there is less TRUM in children with attention-deficit/hyperactivity disorder (ADHD) (see Table 4). From mixed models, LSMeans estimates: *P* values are from diagnosis (ADHD vs. TD) by block (Task vs. Rest) interaction terms; error bars are standard errors of the mean (SEM). See text, Table 4 for regression results.

The categorical diagnosis model evaluated the interactions of diagnosis with task to determine whether reward TRUM differed in ADHD versus TD children. Overall, TRUM was significantly less in ADHD than TD children (task to rest ratio 1.82 in ADHD versus 2.32 in TD, *P *<* *0.0001). Reduced TRUM in ADHD occurred in both the receipt phase (1.80 in ADHD versus 2.36 in TD, *P *<* *0.0001) and the cue phase (1.84 in ADHD versus 2.35 in TD, *P *<* *0.0001). Separating trial types—both reward receipt success/failure, and reward cue red/green/neutral types—produced similar results (see Table 4). See Fig. 5.

### TRUM during reward receipt phase diminished in more hyper/impulsive children

#### Across diagnoses

**Figure 6:**
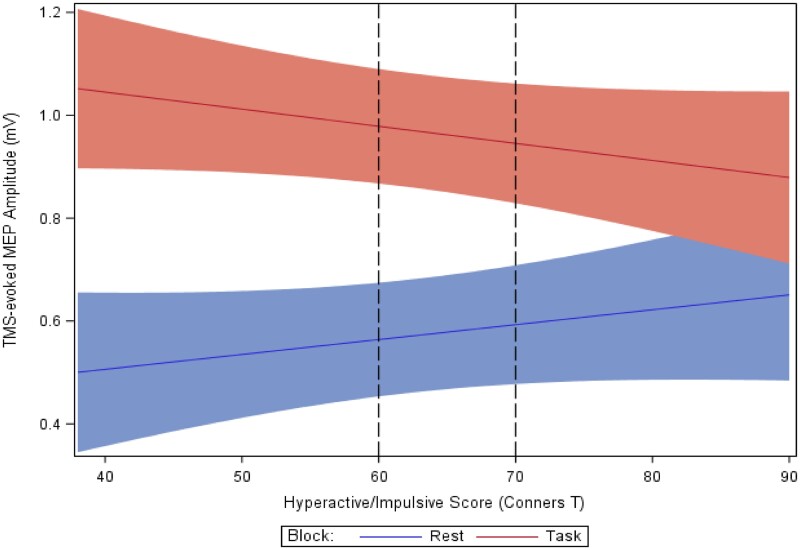
**Task-related up-modulation (TRUM) and hyperactive/impulsive symptom severity.** Motor cortex TRUM during reward receipt phase, successful trials, as a function of attention-deficit/hyperactivity disorder (ADHD) score severity. Less reward-receipt TRUM with greater hyperactive/impulsive symptoms: the lower, solid line blue indicates the TMS MEP amplitudes at rest; 20 rest trials per participant; while the upper solid red line indicates the TMS-evoked MEP amplitude during the outcome, reward-receipt phase of the reward task; approximately 54 trials per participant. TRUM is ratio of task to rest values. Less TRUM is represented by less vertical ‘distance’ from the rest line to the task line. Worse hyper/impulsive scores are statistically associated with less TRUM [*F*(1,4167) = 25.23, *P *<* *0.0001] across the cohort, and also after stratification within TD [*F*(1,1634) = 41.3; *P* = <0.0001] and ADHD [*F*(1,2265) = 6.9; *P *=* *0.009] groups (See Table 4).

To determine whether the reduction in TRUM was greater among children with more severe symptoms, the regression models were re-run with the ADHD Conners Scores. Analyzing trials with TMS pulses during reward-receipt, TRUM was more reduced among children with higher inattentive scores after both successful trial outcomes [*F*(1,3982) = 62.00, *P *<* *0.0001] and failure outcomes [*F*(1,3163) = 44.94, *P *<* *0.0001]. Findings for children with higher hyperactive/impulsive scores were similar for both successful trials [*F*(1,4127) = 86.25, *P *<* *0.0001] (see Fig. 6) and failure trials [*F*(1,3252) = 48.93, *P *<* *0.0001). These differences in TRUM related to symptom severity were not significantly affected by cue type (not shown).

#### Within diagnoses

Regression models were re-run, stratified by diagnosis. For cue phase, more reduced TRUM was marginally associated with higher inattentive scores among TD children during Red trials [*F*(1,1446) = 4.03; *P *=* *0.05]. More reduced TRUM was significantly correlated with higher hyperactive/impulsive scores for TD children for all Cues: Red [*F*(1,1788) = 16.54; *P* = <0.0001], Green [*F*(1,1897) = 13.31; *P *=* *0.0003], and Grey/Neutral [*F*(1,1746) = 7.77; *P *=* *0.005]. No within-diagnoses associations of TRUM and inattentive or hyperactive–impulsive scores were found for children with ADHD during the Cue phase.

Stratifying receipt phase data by diagnosis, within the TD cohort, in children with higher inattentive scores, TRUM was marginally associated in success [*F*(1,668) = 5.96; *P *=* *0.02] and failure outcomes [*F*(1,512)= 3.85; *P *=* *0.05]. No significant associations were present for inattentive scores within the ADHD cohort. Within the TD cohort, higher hyperactive–impulsive scores were highly significantly associated with more reduced TRUM in both success [*F*(1,1634) = 41.3; *P* = <0.0001) and failure outcomes [*F*(1,1254) = 30.89; *P* = <0.0001]. Within the ADHD cohort, higher hyperactive–impulsive scores were significantly correlated with more reduced TRUM after successful outcomes only [*F*(1,2265) = 6.9; *P *=* *0.009].

### Exploratory analyses to address potential predictive or confounding factors

Including in the models, other baseline physiological measures (RMT, AMT, CSP, ICF, MEP amplitudes) did not demonstrate additional statistically significant relationships with SICI or TRUM (not shown).

In analyzing the effect of repeated TMS pulses over time, we found that MEP gradually decreases in both groups throughout the duration of the task (rate of change in ADHD −0.00078 vs. TD −0.00013), but at no time throughout the task was the difference significant. RT was not significantly affected by repeated TMS pulses.

### Exploratory analyses in the absence of motion

To explore if this reward task induced TRUM and reduced SICI in the absence of a requirement for movement to catch the coin, the additional block of non-motion trials was analysed in the same statistical manner used for the main task. That is, MEP amplitudes were the dependent variable and factors of interest were (i) effects of task (TRUM: all reward trials vs. all rest trials) and (ii) effects of PulseType (SICI: PulseType effect during all reward trials vs. all rest trials). Even without a requirement to move, this reward task induced TRUM [Task *F*(1,4693) = 545.58, *P *<* *0.0001] and reduced SICI [PulseType*Task interaction *F*(1,4594) = 5.72, *P *=* *0.017]. Further detailed analyses based on diagnosis and symptom severity, and comparisons of non-movement trials to movement trials, were not performed due to the small number of non-movement trials per participant.

## Discussion

The main, novel findings of this study of ADHD in childhood are that (i) reward task participation, in both the cue phase and the receipt phase, significantly affects both Transcranial Magnetic Stimulation (TMS)-evoked short interval cortical inhibition (SICI) and Task-Related-Up-Modulation (TRUM); (ii) effects of reward on SICI and TRUM differ in children with ADHD versus typically-developing (TD) controls; and (iii) associations of reward-induced M1 changes with symptom ratings are most robust for reduced TRUM during reward receipt, among children with greater hyperactive/impulsive symptoms. In this study, we employed paired pulse TMS to probe modulation of motor cortex (M1) physiology in 105 comprehensively-evaluated 8 to 12-year-old children. We replicated prior studies showing reduced SICI in ADHD in resting M1.^11^^,^^12^^,^^18^ We extended this into a clinically relevant domain of dysfunction in ADHD, using a child-friendly reward game that incorporated the Expectancy Theory of Motivation,^52^ allowing for separate assessments of effects of cueing for varying levels of motivation, and of receipt for both success and failure.^50^

### TMS measures are influenced by reward

Both SICI and TRUM were affected by participation in this reward task in ways that are unlikely to be explainable simply because the task involved movement. It has been shown previously that during a time-window of 100–150 msec prior to movement, SICI is reduced and MEP amplitudes are up-modulated.^16^^,^^53^ However, this reward task (see Fig. 1) probes M1 SICI before (cue) and after (receipt) this time window. Moreover, our exploratory analysis supports that reward modulates SICI and TRUM in the absence of a required movement. Broadly, this study supports the role of M1 in neural circuits related to reward valence, and the use of the TMS in the motor system to identify biomarkers relating to impaired domains of function in ADHD.

### RTs differ by diagnosis, correlate with symptom severity

The premise that our Expectancy Theory of Motivation^52^ reward game induced motivation and a sense of agency were supported, in that the RTs were fastest for red cues and slowest for neutral cues. Compared to their TD peers, children with ADHD responded more slowly across all cue types. However, we did not find evidence that the extent to which the motivational state influenced RTs differed by diagnosis. That is, the speed pattern of Red > Green > Neutral was comparable in both groups of children. However, slower reactions were associated with both more severe ADHD symptoms and smaller TMS-evoked MEP amplitudes during the task. This supports the notion that M1 excitability/up modulation, i.e. TRUM, may reflect mechanisms important for rapid responses, and that these mechanisms reflect ADHD both as a categorical diagnosis and as a dimension of impaired behaviour.

### SICI could be a marker of reward’s ‘normalizing’ effect on impairments in ADHD

As reported in multiple published studies,^12–14^^,^^18^ at rest (baseline), children with ADHD had significantly less SICI. This finding was statistically more robust in a repeated measures design incorporating all trials (See Table 3) than in a method-of-means analysis (Table 1). For both groups, during the reward task, SICI ratios increased, approaching 1.0. This may be conceptualized as a ‘turning down’ baseline inhibitory interneuronal input into M1, or, metaphorically, a pulling back on ‘brakes’ which in the case of children with ADHD may be ‘faulty’ at rest.^54^ Compared to baseline, the reward → task reduction in SICI was less in ADHD; however, this appears to be driven by baseline and not task-related differences in SICI. There were no robust differences in SICI based on cue type or reward receipt. Thus, while this study supports that TMS may be used to probe M1 involvement in neural circuits engaged in reward, other TMS protocols or reward tasks might be required to test specific ADHD hypotheses. However, it is interesting to speculate, since SICI ratios in the two groups converged during reward, that SICI might reflect mechanisms that are impaired in ADHD but that tend to ‘normalize’ in contexts where rewards may be achieved. As shown in Fig. 3, the SICI ratios during the reward task were between 0.6 and 0.8. Thus, a ceiling effect (SICI 1.0) is not a likely explanation.

### TRUM may reflect neural hypo-responsiveness to reward in ADHD

Consistent with our findings evaluating M1 physiology during a Slater-Hammel stop signal, response inhibition task,^18^ participation in the Moneybags reward task induced TRUM, but TRUM was significantly diminished in children with ADHD. Reduced TRUM in ADHD was present in both the cue and receipt phases of the task. Although categorical ADHD-related reductions were similar across cue types, there were intriguing findings related to dimensions of clinical impairment. That is, across and within diagnostic groups, children with higher parent-rated hyperactive/impulsive scores had less TRUM during successful trials. So, among children with the most severe hyperactive/impulsive symptoms, reward-TRUM was most blunted.

Notably, among children who participated in both of these studies, preliminary analysis suggests task correlations (both TRUM and SICI) are weak, and therefore TRUM and SICI across both tasks could create distinct clinical subtypes.^55^

Findings in our study are broadly consistent with prior TMS studies in healthy adults showing that M1 physiology reflects neural circuits active during reward-cueing. For example, one study found that the presence and greater salience of reward increases TMS-evoked MEP amplitudes in healthy adults.^38^ It may be the case that TRUM reflects salience of anticipated rewards, and that salience is reduced in ADHD. It has been suggested that persons with ADHD compensate for reward-cue related neural hypo-responsiveness by increasing reward-seeking behaviour.^22^ However, TRUM during reward tasks may depend in part on the timing of the TMS pulse and the nature of the task.^56^

### SICI not impacted by probability of reward or success/failure in this study

Findings in our study also broadly support studies in adults showing that SICI reflects mechanisms related to reward. For example, a study of SICI in healthy adults using a slot-machine reward paradigm showed that during the anticipation/cue phase SICI was reduced (ratios approached 1.0). However, we did not replicate their finding that SICI changes varied based on reward probability.^57^ Differences with our findings may be related to our sample (paediatric versus adult) or to specific task features (active with a sense of agency in our paradigm^50^ versus passive). Our findings also do not corroborate those from a small cohort of healthy adults in which researchers reported differences in SICI varied with success or failure.^36^ This could be due to spurious findings in their smaller sample, or greater heterogeneity in our larger paediatric cohort.

### Cerebral hypo-responsiveness in ADHD: Comparison with fMRI

Comparing results of this study to similar studies of reward responsivity using other modalities supports the idea of cerebral hypo-responsiveness in ADHD. For example, fMRI studies have reported subcortical (ventral striatum)^22^ (right nucleus accumbens)^58^ hypoactivation in ADHD during anticipation of reward. Other imaging studies have yielded results which might appear contradictory to ours, e.g. studies in adults have showing increased activity of the medial orbitofrontal cortex, bilateral ventral striatum, and left dorsal striatum^59–61^ or occipital cortex and orbitofrontal cortex^62^ in ADHD. A large study using complementary TMS and imaging modalities in children might be required to clarify this issue.

### Study limitations

As with all studies of behavioural diagnoses, there may be misclassification of subjects due to the lack of objective criteria. ADHD has substantial heterogeneity. One study has suggested that ADHD children may have non-overlapping deficits in separate networks of cognitive control, reward processing, and temporal control (timing), indicating that the diagnosis of ADHD could potentially be further subclassified,^63^ which we did not do. However, the direction of bias introduced by imprecise diagnosis or insufficient subclassification would likely be towards the null.

Another limitation in comparing across studies is variable requirements for action. We did query whether movement was required for this task to influence M1 physiology and our findings suggest it may not be. However, interpretation requires caution because there were fewer non-move trials and the non-move block was always performed after the movement blocks, possibly resulting in a priming effect whereby the participants might have imagined moving. A more valid comparison would involve randomization with counterbalancing the order of move versus non-move versions, or simply a parallel design, randomization of ADHD and TD participants to a movement version versus a non-movement version.

### Study strengths

We utilized structured diagnostic interviews and standardized rating scales for all participants at study visits, with diagnostic thresholds for ADHD confirmed with clinical impression. We attempted to systematically address problems with categorical diagnosis by also using rating scale severity, however these scales too are subjective and may obscure important behavioural or neurobiological variability. This study controlled for psychoactive medications at the time of study visit with sufficient two-day washout time, which is important for eliminating confounding effects of stimulants on SICI^14^ or performance.^64^

The Money Bags Reward task was designed such that participants would perceive their actions as necessary for success or failure given the colour cue.^50^ Thus, our paradigm provided a comparable measure of successes and failures that allowed us to examine M1 physiology both at the cue and at the trial feedback timepoints without confounding by performance. The use of TMS as a probe allowed precise temporal analysis of the distinct effects of anticipation and reward success or failure on M1 physiology.

## Conclusions

Here, we report, for the first time, that children with ADHD show reduced TMS-evoked TRUM of motor cortex under the influence of reward and that this reduction specifically after reward success is more significant among children with higher hyperactive/impulsive symptoms. These findings are in keeping with those from prior studies using TMS and overall reveal a pattern of children with ADHD showing reduced inhibitory (SICI) and modulatory (TRUM) physiology in primary motor cortex. By designing TMS-compatible paradigms evaluating several relevant domains of function, differences in SICI and TRUM may provide opportunities to develop biomarkers useful for identifying ADHD subtypes. These subtypes might predict differential responses to behavioural or pharmacological therapies.

## Funding

This research was funded by National Institutes of Health R01 MH095014 and R01 MH085328. J.A.D. received funding from the American Academy of Neurology 2019 Medical Student Research Scholarship. E.M.W. receives funding from the National Institute of Neurological Disorders and Stroke Intramural Research Program.

## Competing interests

S.W. reports receives salary support for consulting for Medtronic, Inc. E.P. receives research support from National Institutes of Health, Autism Speaks, Fragile X Research foundation, and Cincinnati Children’s research foundation. EVP receives past research support from the American Academy of Child and Adolescent Psychiatry and past compensation for consulting for Proctor & Gamble, Eccrine Systems, Inc. and Autism Speaks. He receives book royalties from Springer. E.V.P. has no conflicts of interest with the current manuscript. D.L.G. has received honoraria and/or travel support from the Tourette Association of America/Centers for Disease Control and Prevention, the Child Neurology Society and the American Academy of Neurology. He has received compensation for expert testimony for the U.S. National Vaccine Injury Compensation Program, through the Department of Health and Human Services, He has received payment for medical expert opinions through Advanced Medical/Teladoc. D.L.G. has received research support from the NIH (NIMH) and the DOD. He has received salary compensation through Cincinnati Children’s for work as a clinical trial site investigator from Emalex (clinical trial, Tourette Syndrome) and EryDel (clinical trial, Ataxia Telangiectasia). He has received book/publication royalties from Elsevier, Wolters Kluwer and the Massachusetts Medical Society. All the other authors report no potential competing interest.

## Abbreviations

ADHD =: attention-deficit/hyperactivity disorder
AMT =: active motor threshold
CPRS =: Conners Parent Rating Scale
CSP =: cortical silent period
Dx =: diagnosis
ICF =: intracortical facilitation
LSM =: least square means
M1 =: motor cortex
MEP =: motor evoked potential
ms =: milliseconds
mV =: millivolt
RMT =: resting motor threshold
RT =: reaction time
SD =: standard deviation
SE =: standard error
SEM =: standard error of the mean
SICI =: short interval cortical inhibition
TD =: typically developing
TMS =: transcranial magnetic stimulation
TRUM =: task-related up-modulation
